# Metabolic relay gene of aphid and primary symbiont as RNAi target loci for aphid control

**DOI:** 10.3389/fpls.2022.1092638

**Published:** 2023-01-18

**Authors:** Qian Li, Yu Cheng, Jia Fan, Julian Chen

**Affiliations:** ^1^ College of Bioscience and Resource Environment/Key Laboratory of Urban Agriculture (North China), Ministry of Agriculture and Rural Affairs of the People’s Republic of China, Beijing University of Agriculture, Beijing, China; ^2^ The State Key Laboratory for Biology of Plant Diseases and Insect Pests, Institute of Plant Protection, Chinese Academy of Agricultural Sciences, Beijing, China; ^3^ Ministry of Agricultural and Rural Affairs -Center of Applied Biological International (MARA-CABI) Joint Laboratory for Bio-Safety, Institute of Plant Protection, Chinese Academy of Agricultural Sciences, Beijing, China

**Keywords:** Sitobin miscanthi, Buchnera aphidicola, ilvA, metabolic relay, RNAi

## Abstract

**Introduction:**

Aphids form a stable and mutually beneficial relationship with their primary symbiont *Buchnera aphidicola*, which play an important role in providing the missing nutrients to the host aphid. Based on the genome sequence of wheat aphid *Siotobion miscanthi* and its primary symbiont *Buchnera* that we obtained in our previously study, we identified a metabolic relay gene, *ilvA*, involved in the isoleucine synthesis pathway between aphids and *Buchnera*.

**Method:**

In this study, we identified the location and sequence structure of *ilvA* gene in aphid genome, the expression level in different instars and tissues of aphids, and the effect of reducing *ilvA* expression on the growth and development of aphids by bioinformatics analysis, quantitative PCR, RNAi and bioassay experiments.

**Result:**

Our study showed that *ilvA* was expressed at the highest level in the 2^nd^ instar of the aphid, while the expression of this gene was significantly higher in the aphid bacteriocytes than in other tissues. Notably, this gene is localized on the aphid sex chromosome and remains highly conserved and collinearity across different aphid genomes. Knocking down the expression of *ilvA* reduced the aphid body weight and production. However, the indices of mortality decreased slightly, but were not significantly different, compared to the control.

**Discussion:**

The results show that the relay genes between aphids and their symbionts in the metabolism of essential nutrients have potential roles in the growth and development of aphids, meanwhile, providing target loci and new ideas for RNAi-based aphid green control strategies.

## 1 Introduction

Aphids are important pests that cause significant economic losses in agriculture worldwide. Almost all aphids contain endosymbionts, and one of them, *Buchnera aphidicola* (hereinafter referred to as *Buchnera*), which is present in almost all aphids, provides essential nutrients to the host aphid and therefore called primary symbiont (Baumann, 2005). Additionally, aphids have a variety of secondary symbionts in their bodies, and the significance of secondary symbionts in enhancing the adaptation of aphids to adverse environments has been widely reported (De Clerck et al., 2015; Manzano-Marín et al., 2016; Li et al., 2018; Li et al., 2021). In recent years, it is worth noting the growing number of studies have shown that the function of *Buchnera* not only in providing essential amino acids to the host aphid, but also has potential effects in improving the heat tolerance of aphids (Zhang et al., 2019), revealing the differentiation process (Perreau et al., 2021; Zhang S. et al., 2021) and enhancing their resistance to drugs (Guo et al., 2020). Therefore, exploiting the close and mutually beneficial relationship between aphids and *Buchnera* may produce new ideas for developing green control strategies of aphids.

In China, the grain aphid *Sitobion miscanthi* is one of the most prevalent wheat pests and causes substantial economic losses in agriculture (Li et al., 2021). As genome sequencing technologies continuous upgrading and costs decrease, a large number of insect genomic information continues to be deciphered. Based on our previously published genome information of wheat aphid *S. miscanthi* (LF clone) (Jiang et al., 2019) and its primary symbiont *Buchnera* (Li et al., 2022), making it more convenient to study the nutrient metabolism interaction network between them. Previously, we used genomic information to identify a key relay gene *ilvE*, linking *Buchnera* to aphids in the leucine, isoleucine and valine synthesis pathways. Meanwhile, RNA interference (RNAi) experiment reveals a vital function in three essential amino acid synthesis pathways (Li et al., 2022). However, whether exist other metabolic relay genes are present in the aphid and *Buchnera* nutrient synthesis chains and can be used as candidate target genes for RNAi is still unknown.

Here, we have mined another key gene *ilvA* in the aphid-*Buchnera* relay synthesis of isoleucine pathway through the genomic information obtained in our previous work. Sequence and bioinformatics analysis showed that the *ilvA* gene was highly conserved in different aphid genomes, while the gene expression profile in different developmental stages and tissues of aphids was clarified by qPCR assay. Subsequently, the effects of *ilvA* on aphid life parameters were measured by RNAi experiments. Our results indicate that the *ilvA* gene, which links the aphid and *Buchnera* amino acid synthesis pathways, has an important effect on aphid weight and offspring, all of which suggest that *ilvA* gene can be used as candidate target for RNAi against aphids.

## 2 Materials and methods

### 2.1 Aphid rearing

The strains of *S. miscanthi* used in this study was reared on aphid-susceptible wheat seedlings (*Triticum aestivum* L) in the culture room at 20 ± 1°C with a 75% relative humidity and a light: dark photoperiod of 16: 8 hours. After 10 generations, the aphids were used for the following experiments.

### 2.2 Sequences, gene structure, conserved domain and synteny analysis

The gene structure and conserved domains were analyzed using NCBI Batch CDD-search, and the results were visualized by TBtools (v 1.09857) (Chen et al., 2020). Conserved motifs of the genes were analyzed by the MEME program with the following parameters: classic mode, with the number of repetitions set to zero or one per sequence and the maximum number of motifs identified set to 6. Meanwhile, the location information of gene on aphid chromosome was obtained by genome annotation file, and the results were visualized by TBtools. We downloaded the chromosome-level genome and annotations of *A. pisum* (Li et al., 2020), selected the longest representative coding sequences of each gene and translated the nucleotide sequences to amino acid sequences. Then, MCScanX v1.1 (Wang et al., 2012) was used to identify syntenic blocks of genes between *A. pisum* and the previously published chromosome-level genome of *S. miscanthi*, and the results were visualized byTBtools.

### 2.3 Gene expression analysis between autosome and sex chromosome

Considering that the *ilvA* gene is localized on the aphid sex chromosome, we collected newly emerged winged and wingless adult aphids for transcriptome analysis in order to understand the gene expression on the aphid autosomes and sex chromosome. Total RNA from the winged and wingless aphids were extracted with the total RNA extraction regent kit (Tianmo, Beijing, China) following the manufacturer’s instructions. The quality of the RNA samples was evaluated on a 1% (w/v) agarose gel by electrophoresis and quantified by a Nanodrop 20000 spectrophotometer (DNovix, Washington, DC, United States). And enrichment of mRNA with polyA tails by Oligo (dT) magnetic beads. The obtained mRNA was then randomly interrupted with divalent cations in NEB Fragmentation Buffer, and the library was built for the following Illumina sequencing. In order to ensure the quality and reliability of data analysis, the raw reads were filtered by removing reads with adapters, reads with unidentifiable base information (noted as N) or the low-quality reads (reads with Qphred <= 20 with more than 50% of the entire read length in number of bases). Then, fast and accurate comparison of clean reads with our published reference genome (Jiang et al., 2019) using HISAT2 software (Kim et al., 2015). The differentially expressed genes (DEGs) between the winged and wingless aphids were analyzed. Our sequence data have been deposited in the National Center for Biotechnology information’s Sequence Read Archive, https://www.ncbi.nlm.nih.gov/sra (accession no. PRJNA908645).

### 2.4 Expression profile analysis of *ilvA* gene in aphids

*ilvA* was amplified by PCR from *S. miscanthi* cDNA with the specific primers listed in **Table 1**. The nucleotide sequence of the *ilvA* gene from *S. miscanthi* in this paper has been deposited in GenBank under accession number OQ093134. To quantify the *ilvA* transcript levels in different tissues and different developmental stages of aphids, qRT-PCR was performed with the specific primers listed in **Table 1**. The expression of the *ilvA* gene was normalized to the expression of the aphid housekeeping gene *NADH* (Zhang S. Y. et al., 2021). The amplification efficiency amplified with primers was 100.5 and 99.0% for *ilvA* and *NADH*. All treatments had three biological replicates, and each replicate consisted of three technical replicates.

**Table 1:** Primers used in this study.

Target	Primer	Sequence (5’-3’)	Reference
*ilvA*	*SmilvA*-F	ATGGAAGTCGAAGATCCTTTC	This study
	*SmilvA*-R	TCAAAGAATTTTGGGTAATGGT	
	*SmilvA*-q-F	CAGCCGTGTTGTCTGGTACT	
	*SmilvA*-q-R	TGAAGACGTCGTCTGACAGC	
*dsilvA*	*dsilvA*-F	TAATACGACTCACTATAGGG TCGAGGCCTGCAGGAATTTT	This study
	*dsilvA* -R	TAATACGACTCACTATAGGG GCCTTCCACTACGCACTTCT	
*NADH*	*NADH*-q-F	GATAGCTTGGGCTGGACATATAG	Zhang S. Y. et al., 2021
	*NADH*-q-R	CGAGGAGAACATGCTCTTAGAC	

### 2.5 RNAi assay and aphid bioassay experiment

The molecules of dsRNA targeting *S. miscanthi ilvA* (dsilvA) and the gene sequence of the green fluorescent protein (dsGFP), used as negative control, were synthetized according to the specific primers listed in **Table 1**. Meanwhile, the dsRNA and control were diluted to 500 ng/µl in an artificial diet (20% sucrose), and pure aphid artificial diet was used as the blank control. A total of 500 newly born winged adult *S. miscanthi* were picked from fresh wheat plants. After starvation for 2 hours, 15 active *S. miscanthi* were transferred into each feeding devices, and three replicate tubes were set up. After silencing, *S. miscanthi* individuals were collected at different time after treatment with dsRNA. Then, the surviving aphids were counted to calculated mortality. Additionally, the surviving aphids were used to detect RNAi efficiency by qPCR.

In addition, six aphids per 3 pairs were picked from the artificial device to perform the weight measurement. The average of these 3 pairs of weight values was calculated as one biological replicate per time point. Six biological replicates were examined. Moreover, the number of aphid production at different times of treatment was also counted. Six biological replicates were examined.

### 2.6 Statistical analysis

Differences in gene expression level at different time points of RNAi experiment were tested by one-way analysis of variance (ANOVA) followed by Duncan’s multiple range test using SPSS version 23.0 software (IBM, Armonk, NY, United States).

## 3 Results

### 3.1 *ilvA* gene as the initiator of the isoleucine synthesis pathway in aphids

**Figure 1:**
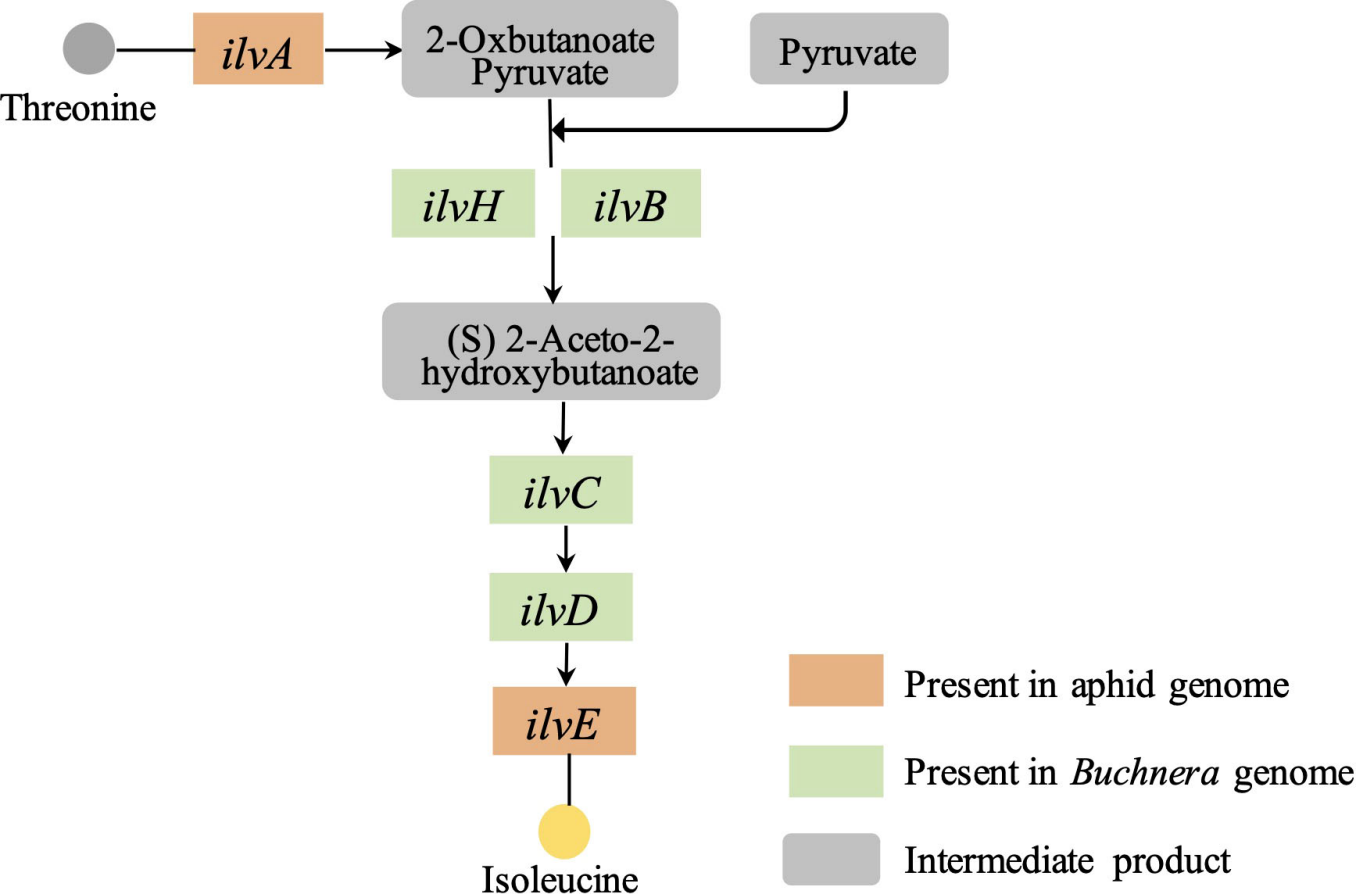
Isoleucine biosynthetic pathways in wheat aphid *S. miscanthi* and *Buchnera*.

Based on our previously reported genome information of the *S. miscanthi* and its primary symbiont *Buchnera* (Jiang et al., 2019; Li et al., 2022), we found that the *Buchnera* genome contains almost all the key genes in the essential amino acid synthesis pathway, however, upstream of the aphid essential amino acid isoleucine synthesis pathway, a threonine dehydratase gene named *ilvA*, which has the function of hydrolyzing threonine to 2-oxybutanoate, is missing from the *Buchnera* genome, but present in the aphid genome (**Figure 1**).

### 3.2 Sequence, structure and phylogenetic analyses of *ilvA* in aphids

**Figure 2:**
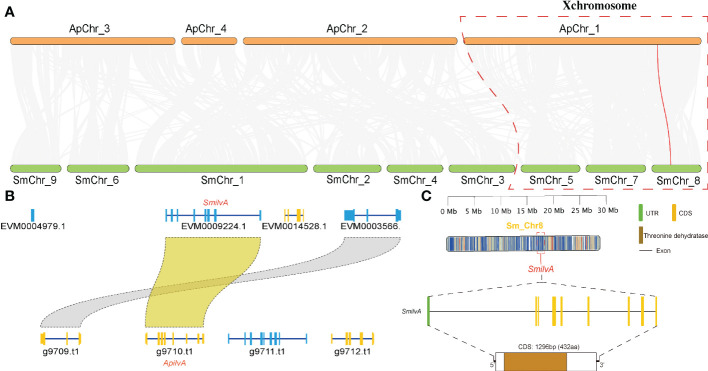
Sequence characteristics, structure and genome collinearity analysis. **(A)** Genome collinearity analysis of wheat aphid *S. miscanthi* and pea aphid *A. pisum.* Red lines represent the *ilvA* gene. **(B)** Colinearity analysis of *ilvA* gene in different aphid genomes. **(C)** Gene structure analysis of *ilvA.*.

**Figure 3:**
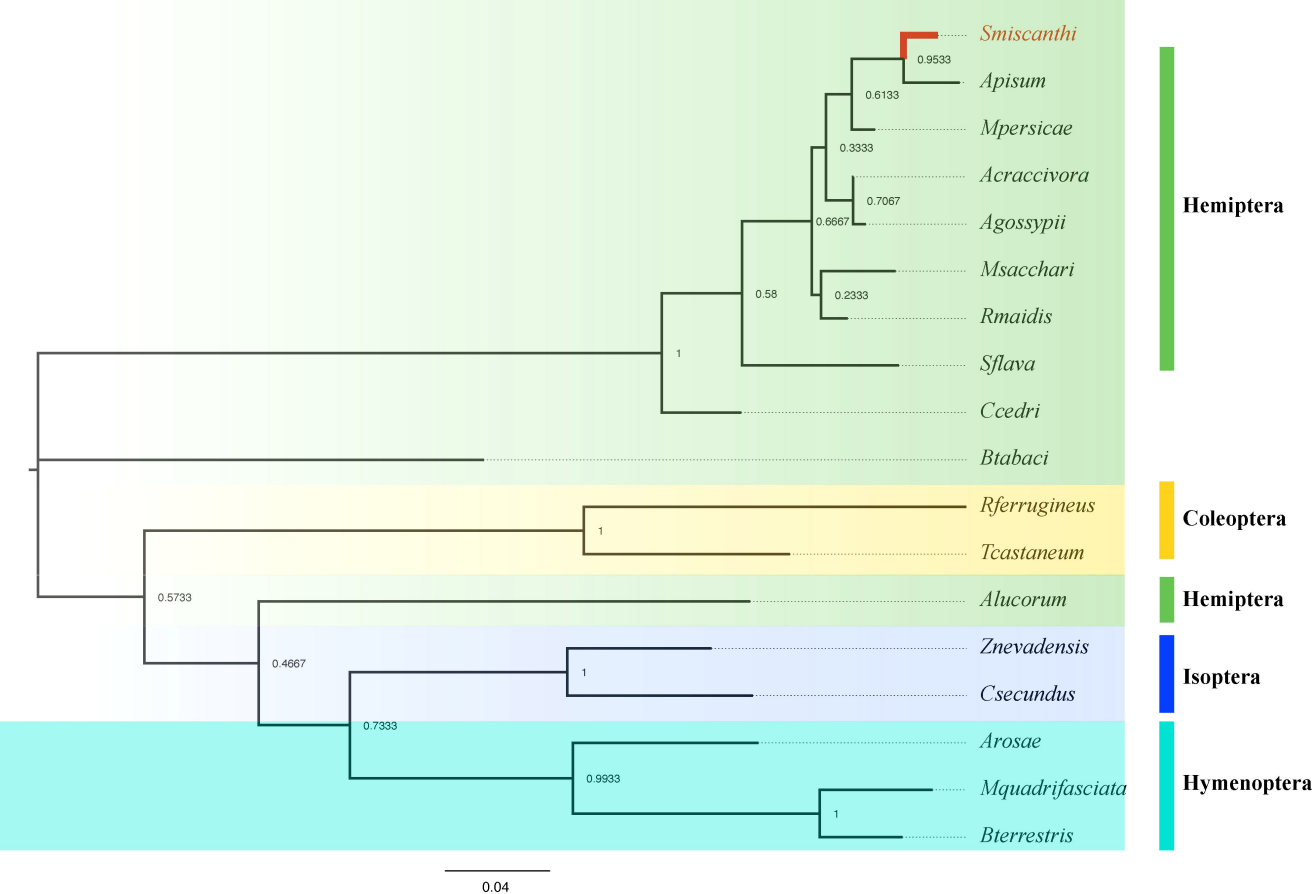
Phylogenetic analysis of *ilvA* gene in different insects.

**Figure 4:**
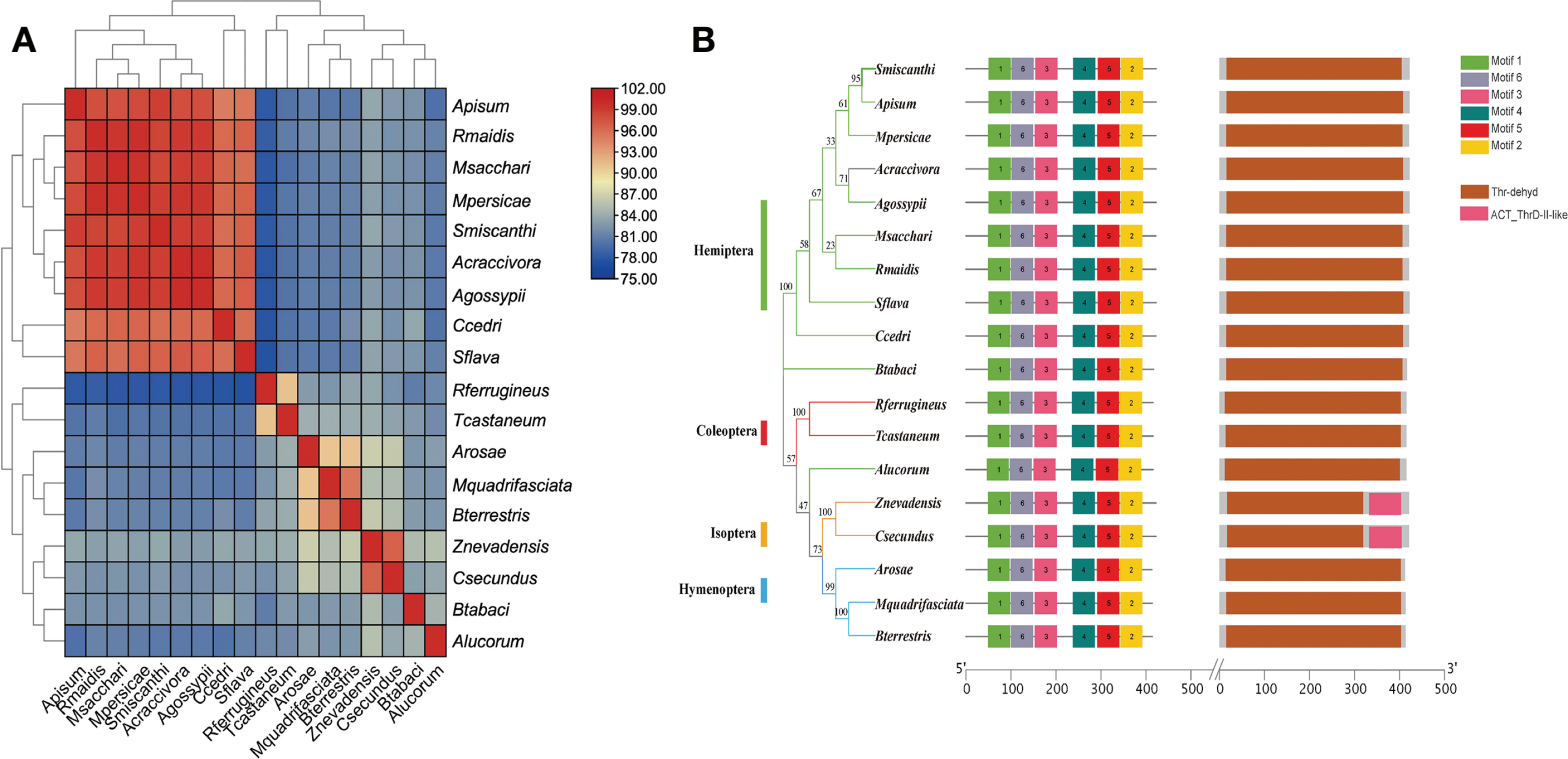
Sequence similarity, motif and domain analysis of *ilvA* gene in different insects **(A)** Sequence homology heat map analysis of *ilvA* gene in different insects. **(B)** Analysis of motifs and domains in *ilvA* gene sequence.

To verify the accuracy of genome sequencing and understand the function of the *ilvA* gene, we cloned the *ilvA* gene using specific primers (**Table 1**). The full-length *ilvA* gene (1272 bp) was obtained by PCR amplification, with GenBank accession number OK431491. The *ilvA* gene is localized on *S. miscanthi* chromosome 8 (SmChr_8), encoding 423 amino acids with a deduced MW of 45.4 kDa and possessing a threonine dehydratase structural domain (**Figure 2**). Interestingly, *S. miscanthi* chromosome 8 is derived from a sex chromosome split that is thought to be highly homozygous and conserved in different aphid genomes (sex chromosome splitting due to chromosome splicing problems during the pre-sequencing process cannot be excluded). Additionally, synteny analysis between the *S. miscanthi* and pea aphid (*A. pisum*) genomes revealed that *ilvA* is also highly conserved in terms of gene location (**Figure 2A**). Phylogenetic analysis showed that *ilvA* gene sequences in different insects clustered into different branches, implying that the gene is highly conserved in different insects. Domain structure and motif analysis showed that *ilvA* genes are highly conserved in Hemiptera, especially in aphids (**Figures 3**, **4**).

### 3.3 Gene expression analysis of different chromosomes and selection pressure analysis of *ilvA* gene

**Figure 5:**
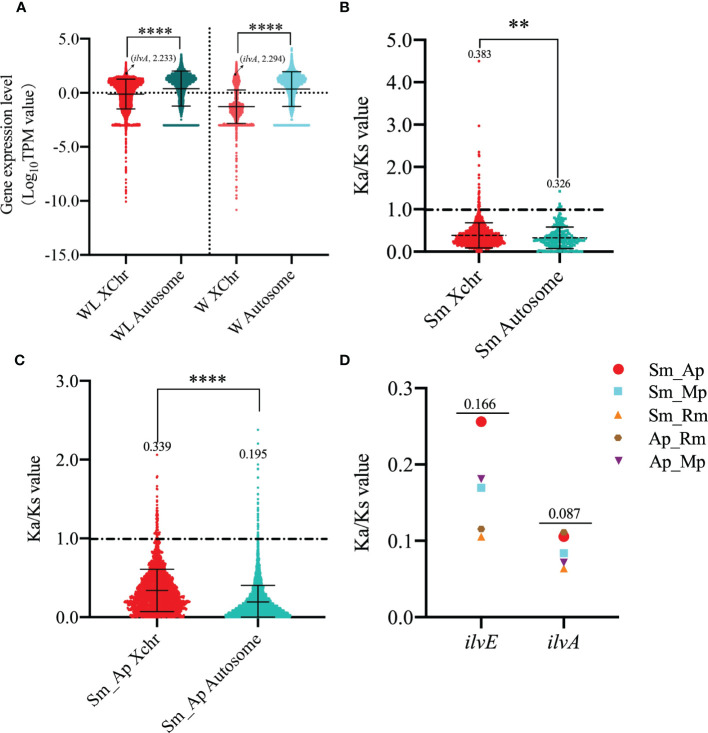
Analysis of gene expression levels and selection pressure on different type of chromosomes in winged and wingless adult aphids **(A)** Analysis of gene expression on different chromosome of winged and wingless adult aphids. **(B)** Analysis of selection pressure of different chromosome paralogous genes in *S. miscanthi.* **(C)** Analysis of selection pressure of different chromosome orthologous genes in *S. miscanthi* and *A*. *pisum.* **(D)** Selection pressure analysis of *ilvA* and *ilvE* orthologous genes in different aphid genomes. The “**” and “****” indicates significant differences based on the Mann-Whitney U test for two sample comparison at P < 0.001 and P < 0.00001, respectively.

Considering the specific location of *ilvA* on the sex chromosome of aphids, and sexually mature aphids are rarely found in *S. miscanthi*, therefore, we sequenced the transcriptomes of winged and wingless adult aphids to investigate the differences in gene expression patterns on the autosomes and sex chromosomes of *S. miscanthi*. Surprisingly, the expression of genes on autosomes was significantly higher than that on sex chromosomes in both winged and wingless adult aphids (**Figure 5A**). To investigate the selective pressure on genes with expression levels in winged and wingless adult aphids, we estimated Ka/Ks value for paralogous genes within the autosome and sex chromosome of *S. miscanthi*, meanwhile, we also estimated Ka/Ks value for single-copy orthologous genes for a pair of related aphid species (*S. miscanthi*/*A. pisum*). The results showed that the selection pressure on sex chromosomes was significantly higher than that on autosomes, whether it was paralogous genes on aphid chromosomes (*p* < 0.0056, Kruskal-Wallis rank sum test) or orthologous genes on different aphid chromosomes (*p* < 0.0001) (**Figures 5B, C**). In addition, the Ka/Ks values of *ilvA* and our previously reported *ilvE* gene in different aphids were very low, 0.166 and 0.087, respectively (**Figure 5D**), suggesting that these genes are under relaxed purifying selection and the function is stable in aphid genome.

### 3.4 Expression profile of *ilvA* at different tissues and developmental stages in *S. miscanthi*

**Figure 6:**
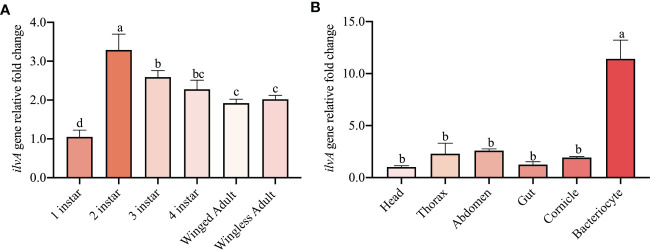
Expression of *ilvA* genes at different tissues and developmental stages in *S. miscanthi* **(A)** Expression level of *ilvA* gene in aphids at different developmental stages. **(B)** Expression level of *ilvA* gene in different tissues of aphid. Different letters above the bars indicate significant differences at P < 0.05.

The expression profile of *ilvA* at different tissues and developmental stages was examined using real-time PCR. Interestingly, the *ilvA* gene was expressed in all instars of aphids, with the highest expression in the 2^nd^ instar and the lowest in the 1^st^ instar (**Figure 6A**). In order to further reveal the expression specificity of *ilvA* gene in different tissues of aphids, we dissected the head, thorax, abdomen, gut, cornicle and bacteriocytes of aphids and performed the qPCR experiment. Unexpectedly, the results revealed that the expression of the *ilvA* gene was significantly higher in the bacteriocytes of aphids than in the other tissues (**Figure 6B**).

### 3.5 Effect of RNAi of *ilvA* on vital parameters of aphids

**Figure 7:**
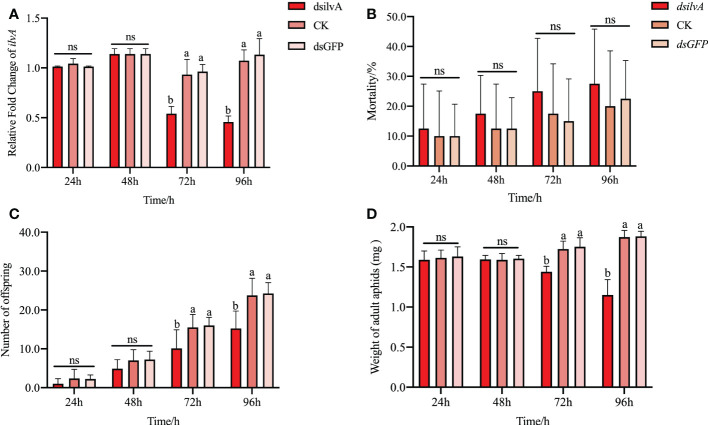
Effect of RNA interference with *ilvA* gene on life parameters of *S. miscanthi* **(A)** Effect of feeding dsRNA at different times on the expression of *ilvA.* **(B)** Effect of interfering with *ilvA* at different times on the aphid mortality. **(C)** Effect of interfering with *ilvA* on offspring of *S. miscanthi*. **(D)** Effect of interfering with *ilvA* on aphid body weight of *S. miscanthi*. Different letters above the bars indicate significant differences at P < 0.05, while ns indicates no significant difference.

To further verify the potential function of *ilvA* in aphid development, we synthesized dsRNA *in vitro* for RNA interference experiments. As shown in **Figure 7A**, the expression level of *ilvA* gene decreased by 46.3% and 54.3%, respectively, after 72 h and 96 h of RNAi treatment (**Figure 7A**). The results showed that the synthesized dsRNA fragments could effectively interfere with the expression of *ilvA* gene. Bioassays showed that compared with feeding dsGFP and sucrose control (CK), feeding dsilvA for 24 and 48 hs had no significant effect on aphid body weight and aphid production, but decreased significantly at 72 and 96 hs (**Figures 7C, D**). Additionally, the indices of mortality decreased slightly, but were not significantly different in all time points, compared to the control (**Figure 7B**). All these results indicate that interference with the *ilvA* gene has a negative effect on the growth and development of aphids.

## 4 Discussion

Aphids and their primary symbiont *Buchnera aphidicola* have formed a long-term and stable symbiotic relationship. At the same time, they are considered to be typical cases in studying the coevolution relationship between insects and endosymbionts (Clark et al., 2000). Numerous studies have shown that *Buchnera* provides aphids with essential nutrients that are missing from the phloem sap of feeding host plants (Shigenobu et al., 2000). There is persuasive experimental evidence that the *Buchnera* genome provides aphids with almost all of the key genes in the essential amino acid synthesis pathway in the model insect pea aphid *A. pisum*, however, little is known about the consistency of this tight nutrient supply chain model in other aphids. Given that almost all aphids contain *Buchnera*, which plays a vital role in aphids, therefore, taking *Buchnera* as the starting point, developing a strategy to break the stable nutrient supply chain between them may become an effective new idea for green prevention and control of aphids. At present, RNA interference technology is widely used in insect gene function verification, is considered to be a new direction for the development of green biological pesticides (Bautista et al., 2009; Belles, 2010; Wuriyanghan et al., 2011; Liu et al., 2020). However, RNA interference technology cannot be effectively implemented in prokaryotes, resulting in direct silencing of aphid primary symbiont *Buchnera* gene is difficult to achieve. Therefore, it has become a new research direction to excavate and identify the relay genes between host aphid and *Buchnera* in the synthesis pathway of essential nutrients and using them as RNA interference target sites. It is gratifying that the availability of the genomes of wheat aphid *S. miscanthi* and its primary symbiont *Buchnera* makes this work feasible.

It has been known for decades that *Buchnera* lacks some genes encoding essential amino acid biosynthesis enzymes and compensates *via* the host pea aphid *A. pisum* (Shigenobu et al., 2000; Wilson et al., 2010). Meanwhile, in our previous study, we found that a metabolic relay gene *ilvE*, a branched-chain amino acid transferase gene required for the final step in the synthesis of the three essential amino acids valine, leucine and isoleucine, was absent in the *Buchnera* genome but was present in the aphid genome (Li et al., 2022). The result was consistent with the previous studies on pea aphid genome (Wilson et al., 2010). In addition, RNAi of the *ilvE* gene significantly increased aphid mortality. Therefore, nutrient synthesis relay genes between aphids and *Buchnera* can be used as candidate targets for RNA interference. However, whether there are other metabolic relay genes in the aphid genome and have potential effect on the aphid development is still rarely reported.

In this study, combined with our previously reported genome data, we identified a threonine dehydratase gene called *ilvA* in the upstream process of isoleucine synthesis, which is absent in the *Buchnera* genome but exists in the aphid genome, and the function of the gene is to hydrolyze threonine to intermediate product 2-Oxobutanoate (**Figure 1**). Therefore, to further verify the function of the gene, we cloned the *ilvA* gene and obtained a complete CDS sequence. Bioinformatics analysis showed that the gene was highly conserved in sequence and location in different aphids (**Figures 3**, **4**). Meanwhile, it is worth noting that this gene is located on the sex chromosome of aphids (**Figure 2A**). In recent years, with the continuous advancement of genome sequencing technology, more and more aphid genomes have been resolved (Chen et al., 2019; Roberto et al., 2020; Zhang S. et al., 2021). Related studies have reported that in different aphid genomes, sex chromosomes are highly conserved, and the selection pressure of genes on sex chromosomes of *A. pisum* is significantly higher than that of autosomes (Li et al., 2020). Moreover, the average expression level of genes on sex chromosomes of pea aphid was significantly lower than that on autosomes, which may be the reason for the significant increase of selection pressure (Jaquiery et al., 2018). In our study, we performed the transcriptome analysis of winged and wingless adult aphids and determined that the expression levels of genes on sex chromosomes were significantly lower than those on autosomes, the results are consistent with previous studies. Moreover, the selective pressure on sex chromosomes was also significantly higher than that on autosomes, whether it was paralogous gene pairs within chromosomes or orthologous gene pairs between different aphids (**Figure 5**). However, the expression level of *ilvA* and *ilvE* gene is much higher than the average value of other genes on sex chromosomes (**Supplementary Table S1**). Meanwhile, *ilvA* and *ilvE* are under low selection pressure in different aphids, which means that the function of this kind of gene in aphid is stable. In general, a lower level of gene expression may imply less important to phenotypes (Nabholz et al., 2013), and previous study implicate that the aphid sex chromosome as a less preferred location for highly expressed genes (Li et al., 2020). However, in this study, we found that although the expression level of genes on sex chromosomes is low overall, there are still some highly expressed genes, and the gene has a potential role in the synthesis of some important nutrients. This result also means that the aphid sex chromosome is still a mysterious region worthy of further study.

To investigate the expression level of the *ilvA* gene in aphids, we next determined the expression pattern of the *ilvA* in different developmental stages and tissues of *S. miscanthi* by qPCR. Our results showed that the expression of the *ilvA* gene was significantly higher in the 2^rd^ instars of aphids than in the other instars and exhibited lowest expression level in 1^st^ instars (**Figure 3**). Surprisingly, the *ilvA* gene was highly expressed in the bacteriocytes of aphids, where *Buchnera* shelters. Interestingly, the specific expression location of this gene is consistent with another synthetic relay gene *ilvE* (Li et al., 2022). Previous reports suggest that *Buchnera* plays an important role in maintaining amino acid synthesis and supply homeostasis in aphids (Wilson et al., 2010). Therefore, with the growth and development of aphids, the increasing titer of *Buchnera* may have a potential role in regulating the expression of *ilvA* and *ilvE*. Moreover, considering the specificity of the expression location of *ilvA* and *ilvE* genes, we hypothesized that the closer distance to *Buchnera* may be more convenient for the synthesis and transport of essential amino acids regulated by aphids between bacteriocytes and hemocoel.

To further study the function of *ilvA* in aphid, the RNAi experiment was performed. Unlike the *ilvE* gene, the indices of mortality decreased slightly, but were not significantly different, after feeding dsilvA 72 and 96 hs (**Figure 7B**). However, the weight and production of aphids decreased significantly (**Figures 7C, D**). Recent studies have shown that common ancestor of Hymenoptera lose all key genes in the valine, leucine and isoleucine synthesis pathway, but parasitoids are able to control related pathways in their host insect, providing them with missing essential nutrients (Ye et al., 2022). Compared with the commensalism relationship between parasitoids and host insects, aphids form a long-term stable mutualistic relationship with their primary symbiont *Buchnera*. The relay synthesis process in the nutrient synthesis pathway is important evidence of coevolution. Therefore, the destruction of synthetic relay chain may be the reason for the negative impact on the growth and development of *S. miscanthi* after knocking down the expression of *ilvA* and *ilvE.* However, there are also relevant study showed that the *Buchnera* protein HisC could functionally replace the missing *ilvE*, catalyzing the terminal reaction in these pathways (Shigenobu et al., 2000). It is worth noting that the *ilvA* gene is the upstream gene of the pathway, which is different from the downstream gene *ilvE* in the synthesis pathway. Therefore, whether reducing the expression of *ilvA* will cause functional complementation or expression response of some genes in the downstream pathway is still needs further exploration. At the same time, reduced expression of the *ilvE* gene significantly inhibited Leu, Ile and Val production in aphids (Li et al., 2022). Although in this study, we did not quantify isoleucine production in aphids after *ilvA* interference, but as a result of our previous studies, we speculated that the decrease in aphid growth fitness may be potentially associated with amino acid production, meanwhile, the decrease in the production of single one essential amino acid may also have a much smaller negative effect on aphids than the three essential amino acids. Such speculation also provides an explanation for why silencing the *ilvA* gene does not result in a significant increase in mortality.

Moreover, secondary symbiont in insect also has potential functions in compensating for the lack of amino acids (Gómez-Valero et al., 2004; Ju et al., 2017). Whether secondary symbiont rescue the negative effects on aphid growth and development caused by RNA interference remains unknown. It is worth noting that *ilvE* and *ilvA* are highly conserved in different aphids, so whether the RNAi targeting them has broad consistency is worthy of further study, which will also provide a theoretical basis for screening broad-spectrum RNAi target sites.

Numerous reports have indicated that almost all insects contain various types of endosymbionts (Baumann, 2005). However, so far, a large number of insect endosymbionts have not been cultured *in vitro*, making the study of their function dependent on host insects (Moran and Mira, 2001). Moreover, RNAi technology is not available for prokaryotic endosymbiont-associated genes, making it difficult to realize the strategy of using symbionts to control insects, necessitating new ideas. In this study, based on our previous studies, we identified another highly conserved isoleucine synthesis pathway upstream gene *ilvA* that is absent in the *Buchenra* genome but is present in the aphid genome, which plays an essential role in influencing the body weight and reproduction of aphids. With the growing popularity of genome sequencing, an increasing number of data resources on the genomes of insects and their endosymbionts have been deciphered. Our study may provide a future direction for targeting important junctions in the endosymbiont-insect metabolic relay process to control agricultural pests.

## Data availability statement

The data presented in the study are deposited in the NCBI repository, accession number PRJNA908645.

## Author contributions

QL, JF and JC conceived and designed the experiments. QL and YC performed the experiments. QL analyzed the data. QL and YC wrote the paper. All of the authors read and approved the final version of the manuscript. All authors contributed to the article and approved the submitted version.

## References

[B1] Baumann P. (2005). Biology bacteriocyte-associated endosymbionts of plant sap-sucking insects. Annu. Rev. Microbiol. 59, 155–189. doi: 10.1146/annurev.micro.59.030804.121041 16153167

[B2] Bautista M. A. M. Miyata T. Miura K. Tanaka T. (2009). RNA Interference-mediated knockdown of a cytochrome P450, CYP6BG1, from the diamondback moth, *Plutella xylostella*, reduces larval resistance to permethrin. Insect Biochem. Mol. Biol. 39, 38–46. doi: 10.1016/j.ibmb.2008.09.005 18957322

[B3] Belles X. (2010). Beyond drosophila: RNAi *in vivo* and functional genomics in insects. Annu. Rev. Entomol. 55, 111–128. doi: 10.1146/annurev-ento-112408 19961326

[B4] Chen C. J. Chen H. Zhang Y. Thomas H. R. Xia R. (2020). Tbtools: an integrative toolkit developed for interactive analyses of big biological data. Mol. Plant 13, 1194–1202. doi: 10.1016/j.molp.2020.06.009 32585190

[B5] Chen W. Shakir S. Bigham M. Richter A. Fei Z. J. Jander G. (2019). Genome sequence of the corn leaf aphid (*Rhopalosiphum maidis* Fitch). Gigascience. 8, 1–12. doi: 10.1093/gigascience/giz033 PMC6451198 30953568

[B6] Clark M. A. Moran N. A Baumann P. Wernegreen J. J. (2000). Cospeciation between bacterial endosymbionts (*Buchnera*) and a recent radiation of aphids (Uroleucon) and pitfalls of testing for phylogenetic congruence. Evolution. 54, 517–525. doi: 10.1111/j.0014-3820.2000.tb00054.x 10937228

[B7] De Clerck C. Fujiwara A. Joncour P. Leonard S. Felix M. L. Francis F.. (2015). A metagenomic approach from aphid’s hemolymph sheds light on the potential roles of co-existing endosymbionts. Microbiome. 3, 63. doi: 10.1186/s40168-015-0130-5 26667400 PMC4678535

[B8] Gómez-Valero L. Soriano-Navarro M. Perez-Brocal V. Heddi A. Moya A. Garcia-Verdugo J. M.. (2004). Coexistence of wolbachia with *Buchnera aphidicola* and a secondary symbiont in the aphid *Cinara cedri* . J. Bacteriol. 186, 6626–6633. doi: 10.1128/JB.186.19.6626-6633.2004 15375144 PMC516615

[B9] Guo S. K. Gong Y. J. Chen J. C. Shi P. Cao L. J. Yang Q.. (2020). Increased density of endosymbiotic *Buchnera* related to pesticide resistance in yellow morph of melon aphid. J. Pest Sci. 93, 1281–1294. doi: 10.1007/s10340-020-01248-0

[B10] Jaquiery J. Peccoud J. Ouisse T. Legeai F. Prunier-Leterme N. Gouin A.. (2018). Disentangling the causes for faster-X evolution in aphids. Genome. Biol. Evol. 10, 507–520. doi: 10.1093/gbe/evy015 29360959 PMC5798017

[B11] Jiang X. Zhang Q. Qin Y. G. Yin H. Zhang S. Y. Li Q.. (2019). A chromosome-level draft genome of the grain aphid *Sitobion miscanthi* . GigaScience. 8, 1–8. doi: 10.1093/gigascience/giz101 PMC6701489 31430367

[B12] Ju J. F. Hoffmann A. A. Zhang Y. K. Duan X. Z. Guo Y. Gong J. T.. (2017). *Wolbachia*-induced loss of male fertility is likely related to branch chain amino acid biosynthesis and *ilvE* in *Laodelphax striatellus* . Insect. Biochem. Mol. Biol. 85, 11–20. doi: 10.1016/j.ibmb.2017.04.002 28412513

[B13] Li Q. Fan J. Cheng Y. Hou M. L. Chen J. L. (2022). *ilvE* as a potential RNAi target to inhibit amino acid synthesis to control the wheat aphid *Sitobion miscanthi* . Entomol. Gen. doi: 10.1127/entomologia/2022/1528

[B14] Li Q. Fan J. Sun J. X. Wang M. Q. Chen J. L. (2018). Effect of the secondary symbiont *Hamiltonella defensa* on fitness and relative abundance of *Buchnera aphidicola* of wheat aphid, *Sitobion miscanthi* . Front. Microbiol. 9, 582. doi: 10.3389/fmicb.2018.00582 29651279 PMC5884939

[B15] Li Q. Sun J. X. Qin Y. G. Fan J. Zhang Y. Tan X. L.. (2021). Reduced insecticide sensitivity of the wheat aphid *Sitobion miscanthi* after infection by the secondary bacterial symbiont *Hamiltonella defensa* . Pest Manag Sci. 77, 1936–1944. doi: 10.1002/ps.6221 33300163

[B16] Li Y. Y. Zhang B. Moran N. A. (2020). The aphid X chromosome is a dangerous place for functionally important genes: diverse evolution of hemipteran genomes based on chromosome-level assemblies. Mol. Biol. Evol. 8, 2357–2368. doi: 10.1093/molbev/msaa095 PMC7403619 32289166

[B17] Liu S. S. Jaouannet M. Dempsey D. M. A. Christine J. I. Coustau C. Kogel K. H. (2020). RNA-Based technologies for insect control in plant production. Biotechnol. Adv. 39, 107463. doi: 10.1016/j.biotechadv.2019.107463 31678220

[B18] Manzano-Marín A. Simon J. C. Latorre A. (2016). Reinventing the wheel and making it round again: evolutionary convergence in *Buchnera*-*Serratia* symbiotic consortia between the distantly related lachninae aphids *Tuberolachnus salignus* and *Cinara cedri* . Genome Biol. Evol. 8, 1440–1458. doi: 10.1093/gbe/evw085 27190007 PMC4898801

[B19] Moran N. A. Mira A. (2001). The process of genome shrinkage in the obligate symbiont, *Buchnera* aphidicola. Genome. Biol. 2, research0054.1–research 0054.12. doi: 10.1186/gb-2001-2-12-research0054 11790257 PMC64839

[B20] Nabholz B. Ellegren H. Wolf J. (2013). High levels of gene expression explain the strong evolutionary constraint of mitochondrial protein-coding genes. Mol. Biol. Evol. 30, 272–284. doi: 10.1093/molbev/mss238 23071102

[B21] Perreau J. Zhang B. Maeda G. P. Kirkpatrick M. Moran N. A. (2021). Strong within-host selection in a maternally inherited obligate symbiont: *Buchnera* and aphids. Proc. Nati. Acad. Sci. U. S. A. 118, 1–8. doi: 10.1073/pnas.2102467118 PMC8536349 34429360

[B22] Roberto B. Archana S. Cindayniah J. G. Felicidad F. F. Sam T. M. Glen P.. (2020). A chromosome-level genome assembly of the woolly apple aphid, *Eriosoma lanigerum* hausmann (Hemiptera: Aphididae). Mol. Ecol. Resour. 21, 316–326. doi: 10.1111/1755-0998.13258 32985768

[B23] Kim D. Langmead B. Salzberg S. L. (2015). HISAT: a fast spliced aligner with low memory requirements. Nat. Methods 12, 357–360. doi: 10.1038/nmeth.3317 25751142 PMC4655817

[B24] Shigenobu S. Watanabe H. Hattori M. Sakaki Y. Ishikawa H. (2000). Genome sequence of the endocellular bacterial symbiont of aphids *Buchnera* sp aps. Nature. 407, 81–86. doi: 10.1038/35024074 10993077

[B25] Wang Y. P. Tang H. B. DeBarry J. D. Tan X. Li J. P. Wang X. Y.. (2012). MCScanX: a toolkit for detection and evolutionary analysis of gene synteny and collinearity. Nucleic. Acids Res. 40, e49. doi: 10.1093/nar/gkr1293 22217600 PMC3326336

[B26] Wilson A. C.C. Ashton P. D. Calevro F. Charles F. Colella S. Febvay G.. (2010). Genomic insight into the amino acid relations of the pea aphid, *Acyrthosiphon pisum*, with its symbiotic bacterium *Buchnera aphidicola* . Nucleic. Acids Res. 19, 249–258. doi: 10.1111/j.1365-2583.2009.00942.x 20482655

[B27] Wuriyanghan H. Rosa C. Falk B. W. (2011). Oral delivery of double-stranded RNAs and siRNAs induces RNAi effects in the potato/tomato psyllid, *Bactericerca cockerelli* . PloS One 6, e27736. doi: 10.1371/journal.pone.0027736 22110747 PMC3218023

[B28] Ye X. Xiong S. J. Teng Z. W. Yang Y. Wang J. L. Yu K. L.. (2022). Genome of the parasitoid wasp *Cotesia chilonis* sheds light on amino acid resource exploitation. BMC Biology. 20, 118. doi: 10.1186/s12915-022-01313-3 35606775 PMC9128236

[B29] Zhang S. Gao X. K. Wang L. Jiang W. L. Su H. H. Jing T. X.. (2021). Chromosome-level genome assemblies of two cotton-melon aphid *Aphis gossypii* biotypes unveil mechanisms of host adaption. Mol. Ecol. Resour. 00, 1–15. doi: 10.1111/1755-0998.13521 34601821

[B30] Zhang B. Leonard S. P. Li Y. Y. Moran N. A. (2019). Obligate bacterial endosymbionts limit thermal tolerance of insect host species. Proc. Nati. Acad. Sci. U. S. A. 116, 24712–24718. doi: 10.1073/pnas.1915307116 PMC6900525 31740601

[B31] Zhang S. Y. Zhang Q. Jiang X. Li Q. Qin Y. G. Wang W. K.. (2021). Novel temporal expression patterns of EBF-binding proteins in wing morphs of the grain aphid sitobion miscanthi. Front. Physiol. 12. doi: 10.3389/fphys.2021.732578 PMC8427609 34512400

